# The impact of changes in medical school admission procedures on study success: A comparative analysis at Hannover Medical School

**DOI:** 10.3205/zma001751

**Published:** 2025-04-15

**Authors:** Stefanos A. Tsikas, Volkhard Fischer

**Affiliations:** 1 Hannover Medical School, Dean of Studies Office – Academic Controlling, Hannover, Germany

**Keywords:** medical studies, student selection, study success, professional experience

## Abstract

**Background:**

In the academic year 2020/21, alterations were introduced in the admission procedures for medical studies, particularly within the selection quota (AdH). These changes reduced the significance of school-leaving grades (the Abitur) in favor of the Test for Medical Studies (TMS) and considerations of professional training & voluntary service as non-cognitive criteria. The waiting time regulation (WQ) was replaced by a “Special Aptitude Quota” (ZEQ), where experienced professionals were classified based on TMS results. This article examines whether and how those changes have influenced study success in the first two years of medical studies.

**Methods:**

We compare the cohorts of 2020 and 2021 (new admission procedure) with the preceding three cohorts, admitted through the old admission process at Hannover Medical School (MHH). Dimensions of study success include dropout rates, progression in studies (completion of the first section within the standard study period), and performance in all written module examinations during the first two study years. The quota of high school top performers (AQ) serves as the reference group. Using ANOVA and comparative statistics, we investigate changes within and between quotas.

**Results:**

Alterations in admission procedures resulted in ZEQ and AdH cohorts being admitted with significantly poorer Abitur grades. While dropout rates decreased in all considered quotas, it is not statistically significant. ZEQ students were more likely to complete the first section on time compared to WQ. AdH entrants after 2020 achieved significantly higher scores in examinations than cohorts from 2017-2019, closing the gap with high school top performers. Both groups consistently outperform WQ/ZEQ in examinations.

**Discussion:**

Historically, Abitur grades have been a reliable predictor of study success. However, recent years have seen an inflation of excellent high school diplomas. We have demonstrated that a shift away from the Abitur toward aptitude tests and even explicitly non-cognitive criteria does not jeopardize success in medical studies. On the contrary, our findings suggest a trend toward increased study success among ZEQ and AdH. The gap towards AQ, however, remains sizeable.

## Zusammenfassung

**Hintergrund:**

Zum Studienjahr 2020/21 griffen Änderungen in den Zulassungsverfahren zum Medizinstudium, die im Auswahlverfahren (AdH) der Medizinischen Hochschule Hannover (MHH) eine reduzierte Bedeutung der Abiturnote zugunsten von TMS und Berufsausbildung sowie geleisteten Diensten als nicht-kognitive Kriterien zur Folge hatte. Die Wartezeit-Regelung (WQ) wurde durch eine „Zusätzliche Eignungsquote“ (ZEQ) ersetzt, in der die Abiturnote als Kriterium entfällt und Berufserfahrene gemäß TMS-Ergebnis eingestuft wurden. In diesem Artikel wird untersucht, ob und wie sich diese Änderungen auf den Studienerfolg in den ersten zwei Jahren des Medizinstudiums ausgewirkt haben.

**Methoden:**

Verglichen werden die Kohorten 2020 & 2021 (neues Zulassungsverfahren) mit den letzten drei Jahrgängen, die über das alte Zulassungsverfahren an die MHH gekommen sind. Als Dimensionen von Studienerfolg betrachten wir Abbrüche, Studienfortschritt (wurde der 1. Abschnitt in Regelstudienzeit beendet?) und Prüfungserfolg in allen schriftlichen Modulprüfungen der ersten beiden Studienjahre. Die Quote der Abiturbesten (AQ) verwenden wir als Referenzgruppe. Mit ANOVA und komparativen Statistiken untersuchen wir Veränderungen innerhalb der Quoten, aber auch, ob sich die Änderungen auf die Unterschiede zwischen den Gruppen ausgewirkt haben.

**Ergebnisse:**

Die Änderungen im Zulassungsverfahren haben zur Folge, dass ZEQ und AdH mit signifikant schlechteren Abiturnoten immatrikuliert werden. In allen betrachteten Quoten sinken die Abbruchquoten, jedoch nicht signifikant. ZEQ beenden den 1. Abschnitt deutlich häufiger pünktlich als WQ. Die ab 2020 Zugelassenen in AdH erreichen signifikant höhere Punktzahlen in Prüfungen als die Kohorten 2017-2019 und schließen zu den Abiturbesten auf. Beide Gruppen schneiden stets besser in Prüfungen ab als WQ/ZEQ.

**Diskussion:**

Die Abiturnote als Auswahlkriterium ist historisch ein guter Prädiktor für Studienerfolg, jedoch gibt es in den letzten Jahren eine Inflation exzellenter Schulabschlüsse. Wir haben gezeigt, dass eine gewisse Abkehr vom Abitur hin zu Studierfähigkeitstests und sogar klar nicht-kognitiven Kriterien Studienerfolg im Medizinstudium nicht gefährdet. Ganz im Gegenteil finden wir eine Tendenz hin zu mehr Studienerfolg bei ZEQ und AdH. Der Abstand zu AQ bleibt jedoch beträchtlich.

## 1. Introduction

### 1.1. Background

The number of available study places in human medicine and dentistry in Germany is determined based on the admission capacity, thus available academic personnel and patient numbers. These regulations are intended to ensure the fundamental right to free choice of profession in publicly funded degree programs, while also balancing the quality of education and its associated costs [https://www.gesetze-im-internet.de/_appro_2002/BJNR240500002.html], [http://www.schure.de/22220/kapvo.htm]. However, the legal frameworks established for this purpose are not always free of contradictions [1]. As a result, they are subject to ongoing changes, which can, in turn, impact the structure and success of academic programs.

Despite the considerable investment in personnel and financial resources compared to other courses of study, the number of available places still lags far behind the demand from prospective students. As the state medical faculties are obligated to ensure the maintenance of public health care in the future, the selection of the most suitable applicants and a low dropout rate are of great importance. Since the design and structure of medical programs are heavily regulated by legal requirements, they have received relatively little attention, although there is evidence for a considerable influence of the study program design on study success [2], [3], [4].

The effects of various selection procedures on study success, mostly limited to the first section of the Medical Licensing Examination, have been widely investigated [5], [6], [7], [8]. Traditionally, in student selection in Germany, school-leaving grade (Abitur) [9], [10], [11], [12], aptitude tests [13], [14], [15], and selection interviews [16], [17], [18], [19] play the most significant role, although they are differently suited for selecting the best students.

The variety and importance given to selection procedures are explained by the complexity of medical studies, high costs per study place, and the critical role of physicians in healthcare. Students entering the program are expected to have a high likelihood of completion without many delays. As a general rule, the better the Abitur grade (or the TMS), the more likely students are to meet these demands, resulting in fewer dropouts, shorter study time, and better grades [5], [7], [8], [9], [10].

According to the latest ruling of the Federal Constitutional Court [https://www.bundesverfassungsgericht.de/SharedDocs/Entscheidungen/EN/2017/12/ls20171219_1bvl000314en], aptitude has to be the decisive criterion for the allocation of limited study places by state institutions also from a legal-formal perspective. “Aptitude” is measured based on the requirements of the specific course of study and the typically subsequent professional activities, which can be highly diverse in the case of human medicine [8], [20]. Therefore, study places must not be allocated solely based on cognitive criteria; this would violate the fundamental right to free choice of profession. With its ruling, the Federal Constitutional Court declared the decades-long practice of allocating study places, in particular the waiting list rule, as partially unconstitutional, leading the states to amend the regulations of the allocation of study places with new regulations from 2020 onwards [12], which we discuss below.

Regarding the subsequent question of how changes in selection criteria affect study success in an unchanged curriculum, there is scarce data available. This is where our study comes in and presents initial results on the impact of the constitutional court’s ruling through a comparative study at the Hannover Medical School (MHH).

### 1.2. Admission quotas

Until 2019, following the allocation of spots through advance quotas (Non-EU foreigners, military doctors, hardship cases, and second-degree students), 60% of available places at MHH were distributed based on a combination of Abitur grade and selection interview (AdH quota). Within this admission procedure, applicants were pre-selected based on their Abitur. All invited candidates were then ranked based on a weighted score composed of 51% Abitur grade and 49% interview score. Thus, the Abitur dominated formally, which was a political mandate at the time, but the ranking of the candidates was determined by their interview performance [19].

While the MHH directly influenced the criteria used for the AdH quota, places for the best school graduates (AQ quota) and spots on a waiting list (WQ) were centrally distributed. AQ grants direct admission based on an outstanding Abitur. Applicants whose grades were not sufficient for AQ or participation in the selection procedure were placed on a waiting list. The poorer the Abitur grade, the longer the waiting time, extending to at least seven years for recent cohorts. Many applicants on the waiting list began vocational training in the medical sector, typically as nurses or paramedics.

**Figure 1:**
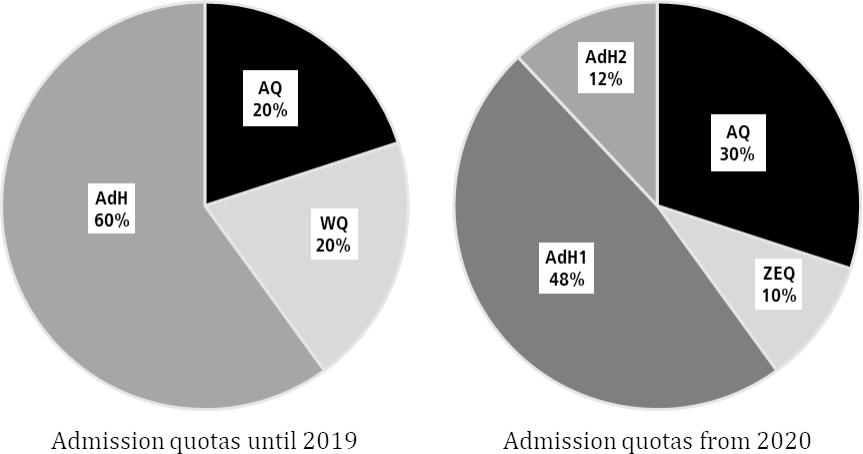
Student admission quotas at MHH Notes: AQ – study places are centrally allocated to the applicants with the best Abitur grade. AdH (university-specific selection procedure) – until 2019, AdH at MHH combined a selection interview and the Abitur grade. Since 2020, admissions via AdH1 were determined by 50% Abitur grade, 30% result of a study ability test (TMS) and 20% voluntary civil service. In AdH2, the dichotomous 20%-criterion is a completed voluntary training in a medically relevant field. WQ (waiting time quota) – according to their Abitur grade, unsuccessful candidates were placed on a waiting list. ZEQ (special aptitude quota) – at MHH, it combines 60% for completed voluntary training and 40% for the result of a study ability test (TMS).

The conclusion of the process depicted in the left image of figure 1 (Fig. 1) occurred after a landmark court ruling deemed the waiting time regulation unconstitutional, calling for greater comparability and standardization of admission criteria [https://www.bundesverfassungsgericht.de/SharedDocs/Entscheidungen/EN/2017/12/ls20171219_1bvl000314en]. The selection based on Abitur grades was in general validated by the ruling. However, non-cognitive criteria must still be considered in each selection process, and the Abitur must no longer be selection-relevant in a sub-quota.

Starting in 2020, the court ruling led to an increase in the AQ quota from 20% to 30% of available places. The WQ quota was replaced by a “Special Aptitude Quota” (ZEQ) now representing 10% of the study positions. In contrast to WQ, criteria for the ZEQ are controlled by the universities. At the MHH, this quota is reserved for individuals with professional experience (in the medical sector). The ranking of these candidates is determined by their performance in an aptitude test; Abitur grades play no role.

In the AdH quota, universities retained some freedom to choose the selection criteria. The MHH discontinued its selection interviews. Instead, the ranking list determining admission was formed from 50% Abitur, 30% aptitude test, and 20% for a non-cognitive criterion. In the AdH2 sub-quota (see figure 1 (Fig. 1)), this non-cognitive criterion is a completed vocational training in the medical field, and in the AdH1 sub-quota, it is a completed voluntary (military, civilian, or social) service. The 20% weighting for a binary criterion resulted in practically all admitted students having completed vocational training or a voluntary service.

### 1.3. Research objectives

Tests of academic aptitude, such as the TMS, reflect different cognitive abilities compared to the Abitur grade; thus, a higher weighting can lead to a shift in the student body composition [21] and potentially affect academic success. Kadmon and Kadmon [13] showed that students with average Abitur grades but high TMS scores achieve better preclinical results than those with excellent Abitur grades and moderate TMS performance. The increased emphasis on the TMS (particularly within the ZEQ) suggests a similar trend might be expected at MHH. Therefore, our aim is to investigate how student Abitur grades evolved under the new admissions procedures and whether these changes impact academic success within and across the AQ, AdH, and WQ/ZEQ quotas.

As regulations for the AQ have remained unchanged, while students admitted via this quota have been subjected to the same general fluctuations in curricula or examination requirements, we treat AQ as a reference group in our analyses.

## 2. Data and methods

### 2.1. Setting

For this study, we analyze data from the first two years of study within the model study program “HannibaL” at MHH. The first two years of study comprise of teaching modules in a preparatory course, cell biology, anatomy, chemistry & biochemistry, physics & physiology, medical sociology & psychology, human genetics and diagnostic methods. These modules place a focus on scientific, medical and clinical fundamentals. In HannibaL, some of the courses include bedside teaching and clinical topics of varying degree that are introduced earlier than in regular study programs at German medical schools. Most modules are graded with Multiple Choice (MC) exams; anatomy and physiology additionally include an oral examination. The module “diagnostic methods” is examined with an Objective Structured Clinical Examination (OSCE) at the end of the second academic year. All exams are graded on a scale from 1 (“A”) to 4 (“D”). Failed exams can be repeated two times. Three failed exams in one course may result in a forced de-registration. Including belated students and failed exams, the average grade in the sample we use for this study is 2.23 (SD: 0.67) or 80.89% (SD: 7.45), corresponding to a B-, with a failure rate of about 6%. From the third year onwards, grades at MHH typically range between 1 and 2 (>85% of questions answered correctly in multiple-choice exams), with a failure rate usually below 1%.

Passing all exams results in the “M1-equivalence” (M1*), which is – in the literal sense – equivalent to one major state examination (M1) after the first two years of study in regular study programs [2]. At MHH, students can attend courses in the more clinically oriented phase (starting in the third academic year) without a completed M1*, which is not possible with the regular state examination at the end of the preclinical phase (M1) [3].

### 2.2. Data

We include five cohorts in our study: students who were admitted via the procedure including a waiting list and interviews (2017-2019), and the first two cohorts admitted via the reformed selection quotas (2020-2021). We focus on these cohorts for mainly two reasons: first, by October of 2023, students from the 2021 cohort were expected to have completed M1* within the regular study duration. The 2022 cohort is expected to obtain M1* not before the fall of 2024. Second, the cohorts 2017-2019 are close to the admission quota reforms of 2020, and their data is deemed comparable to the 2020-2021 cohorts, for example with respect to the Abitur, demographic composition and curricular prerequisites. Information on observations per quota and admission period can be found in table 1 (Tab. 1). In this study, we exclude students admitted upfront via special quotas, as they do not partake in the admission procedure sketched in figure 1 (Fig. 1).

### 2.3. Study variables

For our analysis of study success within and across quotas and cohorts, we use three measures, two indicating the presence or absence of success (dichotomous variables), and one indicating the “extent” of study success.

**Figure 2:**
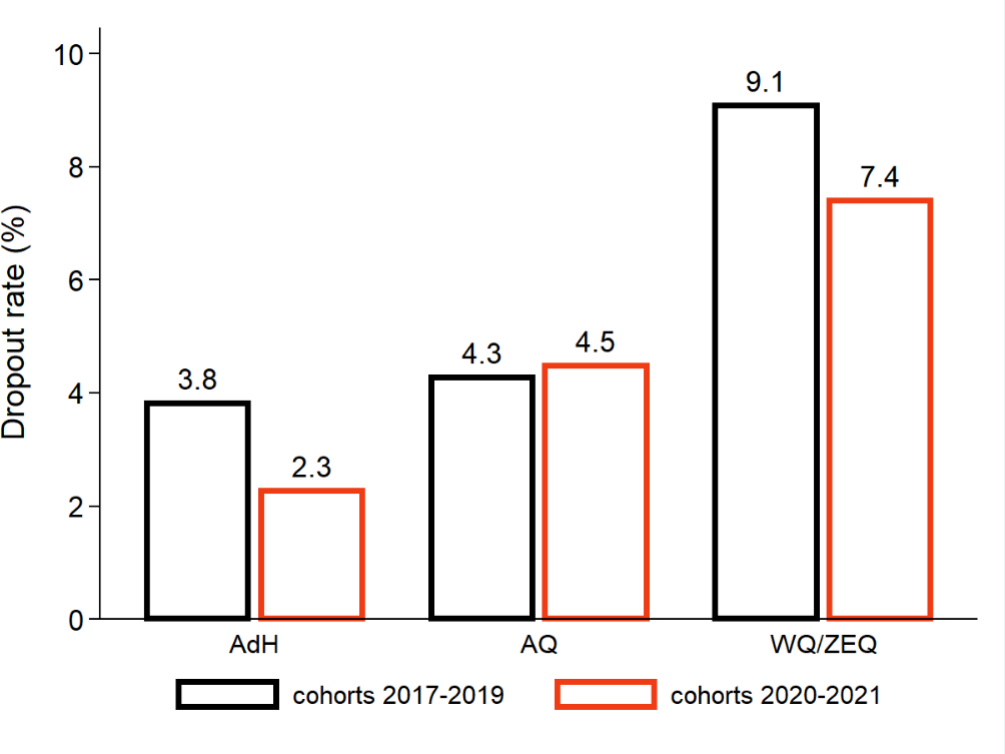
Dropout rate in the three analyzed admission quotas Notes: A dropout is defined as a de-registration due to withdrawal, failed-re-registration, ultimately failed exams, or other unspecified reasons. Motives for leaving university rely on self-declarations. Only the first two years after enrolment are considered to prevent imbalances between cohorts. AQ: numerous clausus quota; WQ: waiting list quota; ZEQ: quota for the professionally experienced; AdH: selection quota, see also fig. 1. Differences in dropout rates within admission quotas are not statistically significant (i.e. *p*≥0.05, two-sided *t*-test).

To analyze the yes/no measures, we initially take a brief, descriptive look at the dropout rate (see figure 2 (Fig. 2)), which we define as de-registrations without M1* and the self-reported reasons “withdrawal”, “failed re-registration”, “ultimately failed exams” and other, unspecified reasons. To prevent imbalances between the cohort 2021 and students admitted in earlier years, we only consider dropouts within the first two years of study. “Older” cohorts have simply more time to fail or withdraw, which becomes more likely with a lack of study progress [3].

However, our focus is on an indicator that tracks timely study progress, thus, whether all written exams (plus the OSCE), collectively referred to as M1* here, were taken and passed within the standard two-year study period from enrollment. This means that all exams must be passed at the first attempt, or at the latest in the retake, typically held a few weeks later, with a minimum grade of 4 (equivalent to a “D”). If an exam (or part of it) is not passed or not taken during this period, the indicator takes the value 0, i.e. the absence of study success in the dimension “study progress”.

This very stringent definition has a valid rationale: our data extends until October 2023, equivalent to a two-year study period for the 2021 cohort. Students from earlier years naturally had more time to pass exams. Tsikas & Fischer [3] demonstrated that particularly WQ students required this additional time and, irrespective of quotas, belated students exhibit significantly poorer exam results. Thus, without imposing a cutoff after two years, we would overestimate the rate of successful M1* completions for cohorts <2021.

For the extent of study success, we use the unweighted mean of the percentage achieved in all written (MC) exams and the OSCE in the first two study years. We only include those students who achieved M1* on time to avoid distortions, as delays correlate with poorer exam performance [3]. Failure to apply this restriction would result in an overestimation of the exam success of the 2020 and particularly 2021 cohorts compared to older cohorts.

Core sociodemographic variables available to us include the school-leaving grade (Abitur), gender, age at enrollment, educational background and the waiting time between school graduation and enrollment at MHH (see table 1 (Tab. 1)).

### 2.4. Empirical strategy

With our analyses, we aim to illustrate how study success has evolved within and between the selection quotas after the change in the admission process. The three groups (AQ, AdH, WQ or ZEQ) and the two different quota systems (pre- and post-2020 procedures) constitute a factorial 2×3 design, analyzed using two-way ANOVA and post-ANOVA comparative statistics. In addition to the main and interaction effects, we control for gender, educational background, and, in a variation, additionally for the Abitur grade and age – factors that constitute the core differences between quotas, particularly with a substantial distinction between AQ and WQ or ZEQ.

**Figure 3:**
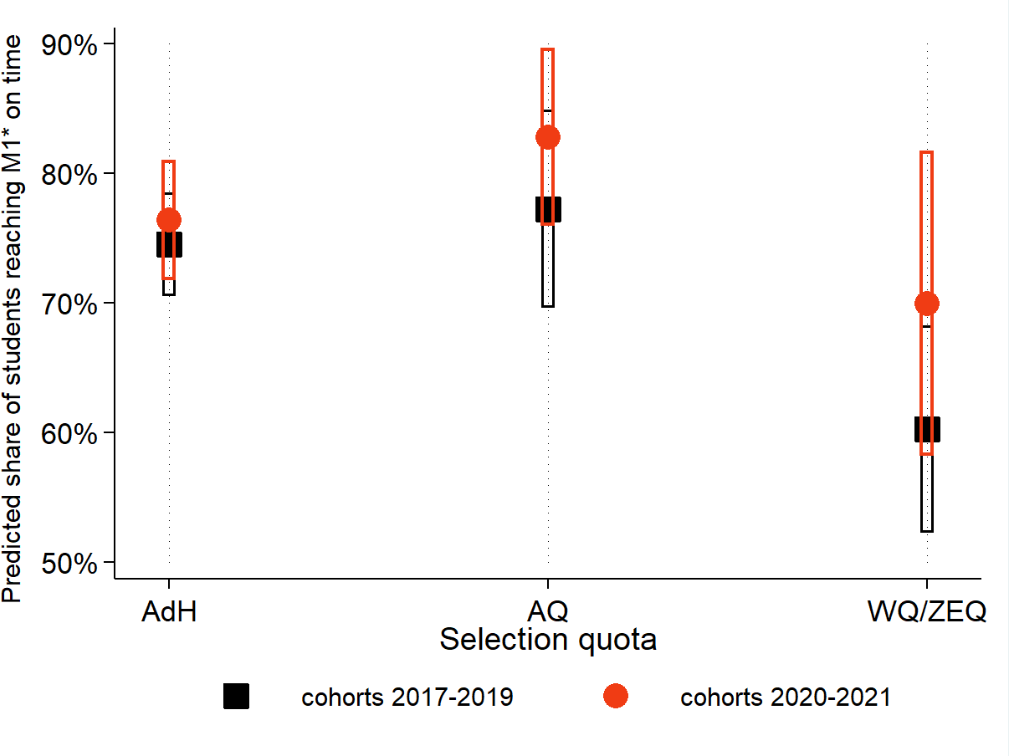
Share of students passing all exams on time, by admission quota Notes: Symbols depict the predicted share of students passing all written exams and the OSCE in the M1*-phase on time, separated by admission quota and cohort-indicator. Fig. 3 is the graphical representation of the interaction term in model (1), tab. 2, and adjusted for gender and school-type. 95% confidence intervals are indicated by bars.

**Figure 4:**
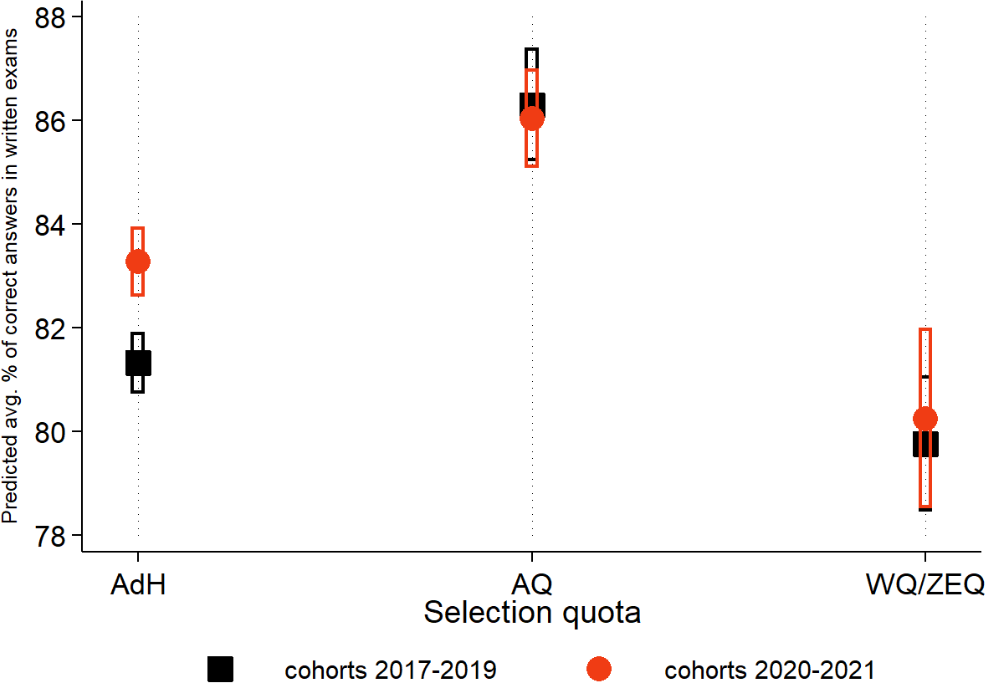
Average success in written exams, by admission quota Notes: Symbols depict the predicted average percent achieved in all written exams and the OSCE in the M1*-phase, separated by admission quota and cohort-indicator. Fig. 4 is the graphical representation of the interaction term in model (3), tab. 2, and adjusted for gender and school-type. 95% confidence intervals are indicated by bars.

However, our interest extends beyond the strength of associations between study success and quotas and admission procedures (ANOVA). We aim to quantify study success and the differences between quotas (before and after procedural changes) in percentage terms. To achieve this, we calculate post-ANOVA partial effects (adjusted for covariates) and present them graphically (see figure 3 (Fig. 3) and figure 4 (Fig. 4)). We define findings as statistically significant when *p*<0.05.

## 3. Results

### 3.1. Sociodemographic statistics

In table 1 (Tab. 1), we show that there are no differences in the sociodemographic composition of AQ students between 2017-2019 and 2020-2021. This finding corroborates our use of AQ as a reference group. Following the alterations in the selection procedure, there have been statistically significant increases in Abitur grades (a higher grade indicates a poorer performance) in the other quotas, rising from 1.3 to 1.5 (AdH) and from 2.4 to 2.6 (WQ/ZEQ), accompanied by moderate to large effect sizes (Cohen’s d).

Within the ZEQ quota, waiting times and, consequentially, the age of entrants have decreased significantly (with moderate effect sizes), a trend likely attributable to the compensatory role of the Test for Medical Studies (TMS) in lieu of the Abitur grade. A noteworthy observation in the WQ/ZEQ quota is a statistically significant 20-percentage point increase in students obtaining their Abitur from a regular Gymnasium (Academic High School).

In the AdH quota, the 20% weight assigned to voluntary services and vocational training has led to a significant increase in the age at enrollment with a moderate effect size, resulting in an average two-year gap between high school graduation and medical school admission. 

MANOVA with the variables included in table 1 (Tab. 1) reveal significant multivariate differences across cohorts for AdH (F(5, 775)=44.38; *p*<0.001) and WQ/ZEQ (F(5, 155)=5.84; *p*<0.001), but not for AQ (F(5, 264)=2.19; *p*=0.056). A two-way MANOVA reveals that the main effect of differences in sociodemographics across quotas (F=685.85; *p*<0.001) markedly trumps the main effect of the change in the admission process (F=15.60; *p*<0.001) and the interaction effect (F=13.31; *p*<0.001).

### 3.2. Dropouts

In figure 2 (Fig. 2), we show that dropouts declined for AdH and WQ/ZEQ following the modifications in the admission procedures. However, these changes are not statistically significant (*t*-test; *p*=0.214 for AdH, *p*=0.719 for WQ/ZEQ). Effect sizes (Cohen’s d) are <0.1 and thus negligible.

Between AQ and AdH, we neither find statistically significant differences in dropout rates for the old admissions procedures (*p*=0.822; *d*=0.023) nor for the cohorts 2020-2021 (*p*=0.175; *d*=0.020). Dropouts among AQ and WQ do also not differ significantly (*p*=0.146), with a small effect size (*d*=0.194). The same applies for the comparison of AQ and ZEQ (*p*=0.409; *d*=0.131). The 5 percentage point difference between AdH and WQ is statistically significant (*p*=0.021) with an effect size of d=0.246. This spread in the dropout rate is also found in the comparison of AdH and ZEQ (*p*=0.038; *d*=0.304).

In all instances, observed dropouts among professionally experienced and waiting list students at MHH are lower than in other study programs [15].

The majority of dropouts occur within the first two years of study, which fits our timeframe covered in this analysis. Tsikas & Fischer [3] reported that WQ students at MHH were enrolled for a longer duration before de-registration. Consequently, dropout rates in this quota (i.e. students who do not graduate) might eventually be 1-2 percentage points above the numbers indicated in figure 2 (Fig. 2). It is too early to make a conclusive statement for ZEQ.

### 3.3. Study progress & exam success

The ANOVA in column (1), table 2 (Tab. 2), reveals a statistically significant, albeit very weak (with respect to F-values and effect sizes) association between admission quotas and timely study progress. The change in the procedure itself and the interaction are not associated with study progress. As shown in column (1), attending a Gymnasium has a moderate association with achieving M1* on time: WQ students have attended a Gymnasium significantly less frequently than AQ and AdH. However, with the transition from WQ to ZEQ, this gap has narrowed (see table 1 (Tab. 1)).

In column (2), we add Abitur grade and age as additional control variables. Particularly age plays a crucial role for timely study progress – older students are more likely to experience delays. These additional variables also affect the other predictors, notably absorbing a substantial portion of the explanatory power attributed to Gymnasium in column (1). The inclusion of these two variables enhances the overall explanatory power of the model. Table A2 in the attachment 1 provides post-ANOVA contrasts for all variables included in table 2 (Tab. 2).

Columns (3) and (4) present the results of the ANOVA with exam success as the dependent variable. Overall, the quotas have a greater influence on exam performance than on timely study progress, in particular the effect size is relatively strong. The change in the admission procedure itself has no impact, and the interaction effect is statistically significant but weak. Attending a Gymnasium is associated with more exam success. Age (column 4) does not play a role here, but the Abitur grade does. It also explains a large portion of the differences between quotas. In summary, quotas, admission procedures, and covariates collectively explain the study success dimension “exam success” better than the dimension “study progress”.

Table A1 in attachment 1 presents descriptive statistics for the three study success measures analogous to table 1 (Tab. 1). 

Figure 3 (Fig. 3) and figure 4 (Fig. 4) illustrate the differences in study progress and exam success between admission quotas and cohorts. Figure 3 (Fig. 3) indicates that students from the WQ quota (cohorts 2017-2019) achieved M1* significantly less frequently on time compared to AQ and AdH. Following the modification of the admission process, the gap between (now) ZEQ and the other quotas has narrowed. A more frequent adherence to the standard study time is observed in all quotas from 2020 onwards. However, the difference compared to 2017-2019 is not statistically significant (as indicated by overlapping 95% confidence intervals). For ZEQ, there is an increase of over 10 percentage points compared to WQ, but due to the small sample size, the confidence intervals are wide.

Figure 4 (Fig. 4) demonstrates that exam success in AQ and WQ/ZEQ remains practically unchanged before and after the modification of the admission procedure. In AdH, we observe a significant increase of approximately 3 percentage points in correct answers on written exams. Both WQ and ZEQ perform significantly worse than AQ and AdH, and the gap with the latter quota has slightly increased since 2020. However, on average, timely ZEQ students still achieve a “good” (B) grade, reaching around 80%. In the years 2017-2019 and from 2020 onwards, AQ outperform their counterparts from AdH. However, this performance gap decreased significantly for cohorts 2020-2021, compared with those admitted until 2019 (see the split-sample analysis in table A3 of the attachment 1 ). A comparison of figure 3 (Fig. 3) and figure 4 (Fig. 4) and the descriptive statistics in table A1 in attachment 1 suggests that the sociodemographic covariates only have a small influence on the study progress and success in exams.

## 4. Discussion

Our analyses of the initial impacts of adjustments in student selection at German medical schools have shown that strengthening the TMS and non-cognitive criteria (at MHH, professional experience or voluntary service) at the expense of the Abitur grade did not negatively affect study success. On the contrary, we find evidence that ZEQ students (TMS determines the ranking) tend to progress more quickly than WQ (Abitur grade determined the ranking). Additionally, the performance gap of ZEQ compared to AQ and AdH is narrower than it was with WQ. In terms of exam success, AdH students admitted after 2019 outperformed their classmates selected through interviews (and Abitur grades) and were also able to catch up significantly with the AQ group.

Our findings also complement another recent study at MHH, which demonstrated that WQ and preliminary quota students who show performance deficits and significant delays in the early phase of their studies (M1*) are able to catch up to some extent with AQ and AdH students by the time they reach M2, although grade differences remain sizeable [3]. The authors of that study concluded that MHH’s program structure, which allows students to enroll in clinical modules even if they have not yet completed M1*, supports this progress.

We suggest that admitting the professionally experiences based on their TMS result instead of the Abitur can further facilitate this adaptation and narrow differences already in the M1*-phase.

Professionally experienced students already possess crucial competencies and practical skills at the time they enroll. This advantage (compared to top high school graduates) becomes evident in assessments of practical and communicative skills, where performance differences across quotas are often absent [22], [23], [24], [25], [26]. However, in regular study programs, where modules with a practical-clinical emphasis are taught only after passing M1, students who fail to overcome this “obstacle” (predominantly the professionally experienced), are not able to play to their strengths.

The Abitur grade as the (sole) selection criterion has been criticized in recent years because particularly excellent grades have increased inflationary, which limits the selectivity of the NC (numerous clausus) criterion, and because a high school diploma in a top-tier range tends to concern a relatively narrow socioeconomic group.

We explicitly argue, however, that our findings do not challenge the validity of the Abitur as a selection criterion; after all, AQ students remain the most successful group overall, based on the parameters for study success available to us. Rather, our results demonstrate that the criteria for selecting the most suitable applicants can be expanded to include dimensions such as medical pre-experience or life experience (through social engagement) without negative consequences for study success, as long as a cognitive study ability criterion such as the TMS is included.

From our perspective, a “diversification” of the student body is an appropriate and worthwhile goal to pursue, as long as the selection of the best candidates is based on criteria associated with success in studies and professional careers. In this sense, efforts on broadening access to medical school should focus on strengthening equality of opportunity before entering the tertiary education sector [25], rather than on the preferential treatment of “disadvantaged” groups in terms of “equality of outcome”, as is sometimes demanded, but to which the authors are highly critical.

A look at the other medical faculties in Germany [26], [27] reveals that most sites responded with adjustments in their selection procedures that are comparable to the MHH. Vocational training, TMS, and civil service or other societal engagements are often considered for AdH and ZEQ, although their weighting differs. More complex instruments such as multiple mini-interviews or personality tests are less common. Overall, the influence of the high school grade has not grown, even though AQ was increased from 20% to 30% nationwide. 

From a broader higher education policy perspective, we see two overarching goals behind the changes in admission procedures. The first is the effort to catch up with international developments of the past two decades (especially in the Anglo-American region or in the Netherlands) towards competency-based medical education, and secondly, the strengthening of general-practice in Germany, which is reflected in the so-called “country doctor quota” (Landarztquote; a pre-quota with a multi-stage selection procedure including Abitur, TMS, vocational training & professional experience, and an interview as criteria).

One limitation of our study is that neither TMS results nor information on vocational training or voluntary service were available to us. While the TMS presumably drives the performance advantage of ZEQ over WQ, this cannot be empirically validated here, as the level of detail in the student statistics at MHH does not allow to identify which AdH students were admitted via sub-quota 1 (voluntary civil service) or 2 (vocational training). For exam success, we had to exclude two oral exams, as they are only graded (on an A-D scale) and were thus not suitable for the empirical approach.

Another limitation could be the prevalent skewness of the grading scale in medical education towards good and very good results, which may be associated with a lack of reliability in the examinations. However, as we have briefly reported in section 2.1, this issue is less pronounced in the first two years of study than in the clinical phase of the program. Especially regarding ZEQ, our study uses a relatively small sample and it must be noted that in 2020 and 2021, waiting time still played a role in the ranking process of ZEQ, albeit with decreasing significance. This gradual transition to the “pure” ZEQ from cohort 2022 onwards may result in even more pronounced differences between WQ and ZEQ than we have observed in our study.

The MHH and other faculties typically established or adjusted quotas without these decisions being informed by students’ study success, and we believe that this study provides valuable information and an ex-post validation. At this point, we can only show evidence for the model study course at MHH. This is mainly driven by the circumstance that only AQ is defined uniformly at all universities. All other quotas vary to a greater or lesser extent between locations due to state- or university specific requirements [26], [27].

Informed by the results presented in this study, the MHH has decided to shift the weighting of the Abitur (from 50% to 30%) to the TMS (from 30% to 50%) in the selection process from 2024 onwards. This step should ensure that applicants with professional experience or a completed voluntary service, who achieved a very good TMS result, have a realistic chance of admission even with an only average Abitur grade and are favored over applicants with a very good Abitur and a mediocre TMS. The reformed quota system thus also aligns with the HannibaL model curriculum, which aims to introduce students to medical practice and patient contact from the first semester, and thus aims to value professional experience and social competencies already in the selection process.

## Notes

### Declarations

In this study, we analyzed only retrospective, administrative and anonymized data. The use of such data for evaluation/research and quality assurance purposes is regulated by sect. 14, para. 1-5 “MHH Immatrikulationsordnung” and sect. 17, para. 3 NHG and rendered a separate approval by an ethics committee unnecessary. Data and code can be made available by the corresponding author upon reasonable request.

### Authors’ ORCIDs

- Stefanos A. Tsikas: [0000-0001-6642-5456]
- Volkhard Fischer: [0000-0001-8499-9437]

## Competing interests

The authors declare that they have no competing interests. 

## Supplementary Material

Supplementary material

## Figures and Tables

**Table 1:**
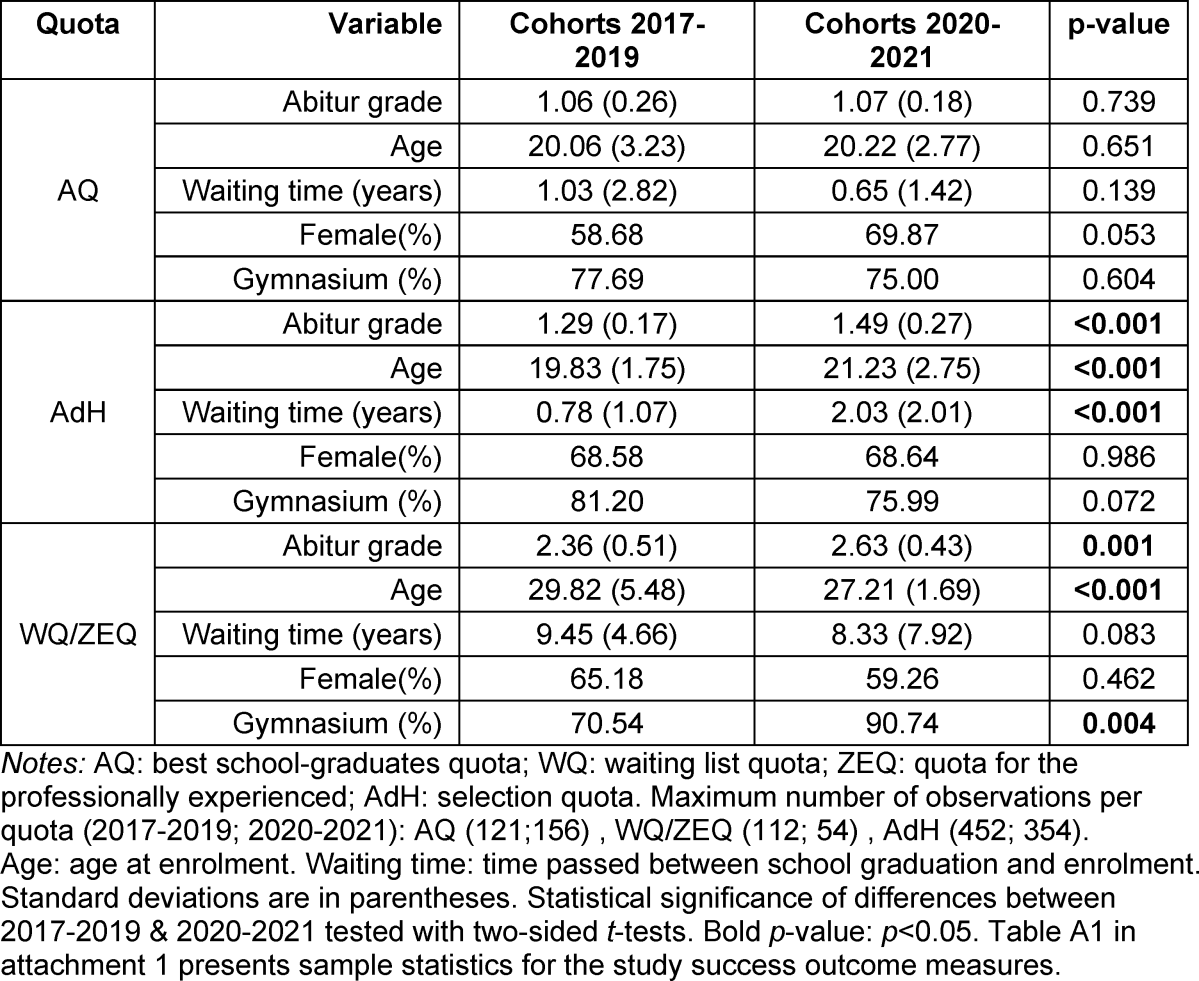
Sociodemographic sample statistics

**Table 2:**
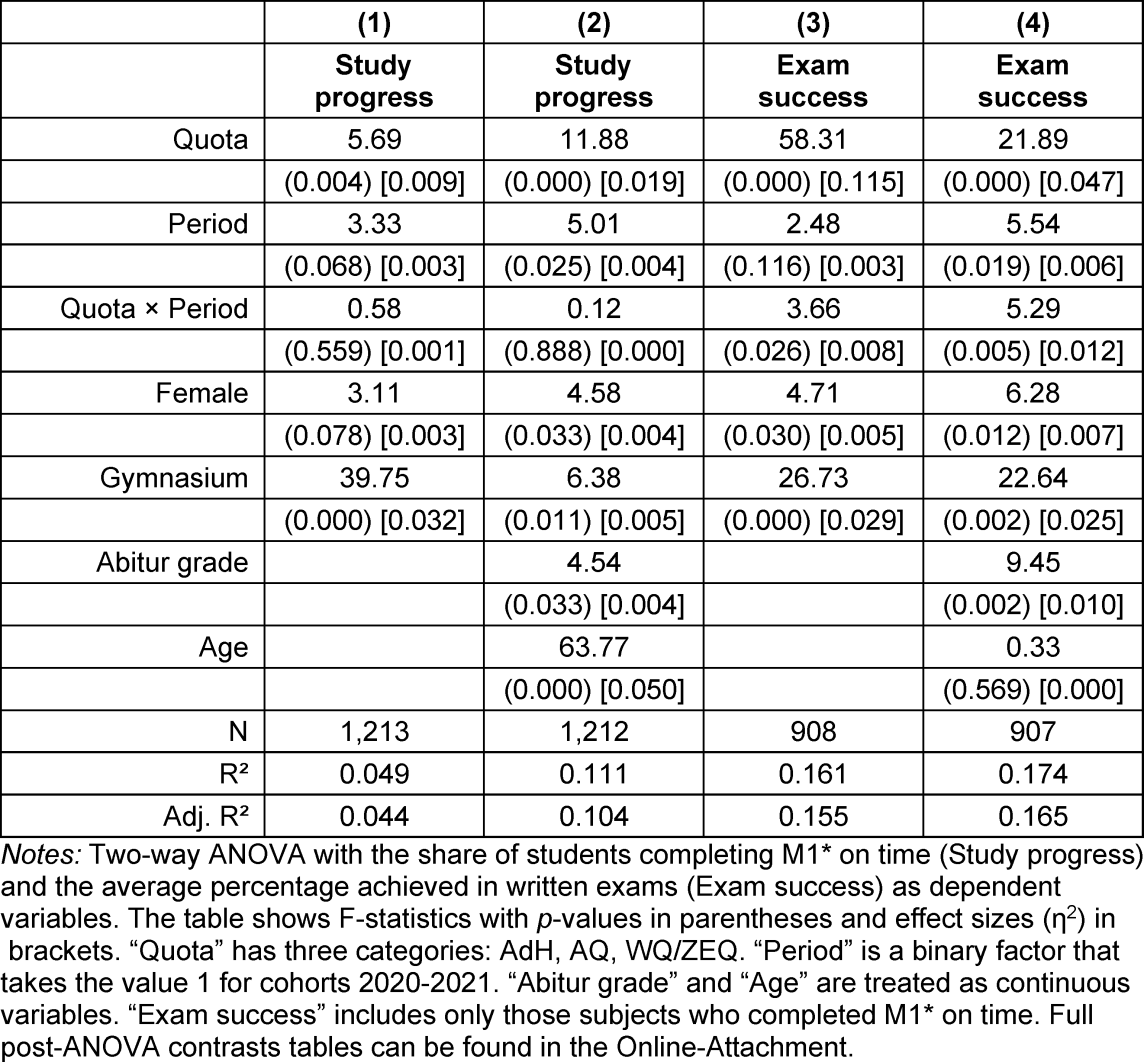
ANOVA results
