# Supplementary material for: The impact of changes in medical school admission procedures on study success: A comparative analysis at Hannover Medical School
Source: GMS J Med Educ. 2025 Apr 15;42(2):Doc27. doi: 10.3205/zma001751 (PMC12131504; doi:10.3205/zma001751)
Supplement: Supplementary material [file JME-42-27-s-001.pdf]

Attachment 1: Supplementary material

Table A1: Descriptive statistics for the study success measures

Quota	Study success (%)	Cohorts 2017-2019	Cohorts 2020-2021	*p*-value	|Cohen's d|
AdH	M1* on time	74.944	76.048	0.723	0.026
	M1* grade	81.338	83.242	**<0.001**	**0.357**
	Dropouts	3.820	2.272	0.214	0.089
AQ	M1* on time	77.500	82.119	0.346	0.115
	M1* grade	86.415	85.923	0.535	0.085
	Dropouts	4.274	4.487	0.932	0.010
WQ/ZEQ	M1* on time	59.091	72.549	0.100	0.280
	M1* grade	79.723	80.506	0.421	0.167
	Dropouts	9.091	7.407	0.719	0.060

Notes: Analogous to Table 1 in the main text, table A1 presents descriptive sample statistics for the three study success measures, defined and explained in section 2.3. Statistical significance of differences within quotas and across selection procedures is tested with two-sided t-tests. Effect sizes are estimated using Cohen's d. Bolded numbers indicate a statistical significant difference/effect.

Table A2: ANOVA contrasts table

		(1) Study progress		(2) Exam success	
**Versus**		Contrasts	S.E.	Contrasts	S.E.
AQ	AdH	0.0459	0.0302	3.8782*	0.4250
WQ, ZEQ	AdH	-0.1034*	0.0391	-2.2884*	0.5889
WQ, ZEQ	AQ	-0.1493*	0.0444	-6.1666*	0.6557
2020-2021	2017-2019	0.0571	0.0313	0.7268	0.4613
**Interactions**					
AdH/1	AdH/0	0.0189	0.0307	1.9517*	0.4408
AQ/0	AdH/0	0.0277	0.0437	4.9842*	0.6216
AQ/1	AdH/0	0.0831	0.0400	4.7240*	0.5573
WQ/0	AdH/0	-0.1425*	0.0452	-1.5571	0.7190
ZEQ/1	AdH/0	-0.0455	0.0628	-1.0680	0.9186
AQ/0	AdH/1	0.0088	0.0452	3.0325*	0.6428
AQ/1	AdH/1	0.0642	0.0416	2.7730*	0.5806
WQ/0	AdH/1	-0.1614*	0.0467	-3.5088*	0.7371
ZEQ/1	AdH/1	-0.0643	0.0640	-3.0197*	0.9334
AQ/1	AQ/0	0.0554	0.0520	-0.2602	0.7283
WQ/0	AQ/0	-0.1701*	0.0561	-6.5413*	0.8568
ZEQ/1	AQ/0	-0.0731	0.0710	-6.0522*	1.0300
WQ/0	AQ/1	-0.2256*	0.0532	-6.2810*	0.8122
ZEQ/1	AQ/1	-0.1285	0.0689	-5.7920*	0.9945
ZEQ/1	WQ/0	0.0970	0.0721	0.4891	1.0926

Notes: Table A2 shows contrasts and standard errors (S.E.) for ANOVA in columns (1) and (3), Table 2 in the main text. The term "/0" following the quota indicator refers to cohorts 2017-2019. The term "/1" refers to the reformed selection procedure since 2020. Table A1 shows the contrasts of all 15 quota-period combinations implicated in the interaction term. *: $p<0.05$.

Split-sample analysis

In Table A3, we check whether the changes depicted in Figures 3 and 4 (main text) between the reference group AQ and the AdH & WQ/ZEQ quotas are statistically significant. To do this, we once again perform ANOVA with the dependent variables “Study Progress” and “Exam Success”, but separately for the cohorts 2017-2019 and 2020-2021. In comparison to the previous two-factor ANOVA, this analysis thus excludes the interaction term and the variable “Period”.

Table A3: Split-sample analysis

	(1) Study progress	(2) Study progress	(3) Wald-test	(4) Exam success	(5) Exam success	(6) Wald-test
	2017-2019	2020-2021	χ^2	2017-2019	2020-2021	χ^2
AdH	-0.026	-0.064	0.46	-5.045*	-2.783*	6.59*
	(0.045)	(0.040)		(0.626)	(0.573)	
**AQ (Reference)**						
WQ/ZEQ	-0.169*	-0.126	0.22	-6.505*	-5.794*	0.33
	(0.058)	(0.067)		(0.862)	(0.984)	
Female	-0.065	-0.021	0.73	-0.021	-1.806*	5.60*
	(0.036)	(0.038)		(0.505)	(0.549)	
Gymnasium	0.185*	0.192*	0.01	2.892*	1.811*	1.39
	(0.041)	(0.043)		(0.646)	(0.669)	

Note: Table A3 presents marginal effects following separate ANOVA for cohorts 2017-2019 and 2019-2020, thus excluding the factors ‘Period’ and the quota-period interaction (see Table 2). Columns (1)-(2): dependent variable is the share of students completing M1* within two years. Columns (4)-(5): dependent variable is the average result (in % correct answers) in written exams of those subjects who have completed M1* on time. Columns (3) and (6) show the results of a Wald-hypothesis test with the null-hypothesis that there are no differences between cohorts 2017-2019 & 2020-2021. The Wald-tests are performed after a Seemingly Unrelated Regression (SUR) of the underlying split-sample ANOVA. Standard errors are in parentheses. *: $p<0.05$.

Table A3 presents post-ANOVA partial effects. The values for AdH and WQ/ZEQ indicate the percentage gap compared to AQ. The model also includes the dichotomous control variables Female and Gymnasium. In column (1), we find that the gap in achieving M1* within the standard study time between AQ and WQ was 17 percentage points and statistically significant. For the cohorts 2020-2021, the disparity between AQ and ZEQ was 12.6 percentage points and not significant. Column (3) presents Wald tests for the null hypothesis that the gaps between AQ and AdH or WQ/ZEQ before and after the adjustment of the selection process remained unchanged. In the case of study progress, we cannot reject this hypothesis for any of the included variables.

In Columns (4)-(6), we repeat the calculations with the continuous dependent variable “Exam Success”. Here, we find that the gap between AQ and AdH in 2020-2021 has statistically significantly decreased by approximately 2.3 percentage points. However, as shown in Figure 4 (main text), the gap between AQ and AdH remained significant. For the comparison of AQ with WQ/ZEQ, we do not observe a significant change. Lastly, compared to men, women performed significantly worse in 2020-2021 than in 2017-2019, with a gap of just under two percentage points.
